# Long-Chain Alkylphenol Biodegradation Potential of Soil Ascomycota

**DOI:** 10.1134/S0012496623700515

**Published:** 2023-10-13

**Authors:** I. L. Kuzikova, N. G. Medvedeva

**Affiliations:** grid.513158.b St. Petersburg Federal Research Center of the Russian Academy of Sciences (SPC RAS), 199178 St. Petersburg, Russia

**Keywords:** Ascomycota, reduced glutathione, degradation, malondialdehyde, nonylphenol, oxidative stress, octylphenol, *Fusarium solani*

## Abstract

A total of 11 ascomycete strains destructing technical nonylphenol (NP) and 4-tert-octylphenol (4-t-OP) were isolated from NP-contaminated soddy-podzolic loamy soil (Leningrad Region, Russia). The isolates proved capable of degrading NP and 4-t-OP at a high load (300 mg/L). The most efficient *Fusarium solani* strain 8F degraded alkylphenols (APs) both in cometabolic conditions and in the absence of additional carbon and energy sources. A decrease in APs was due to biodegradation or biotransformation by the strain and, to a minor extent, absorption by fungal cells. NP and 4-t-OP half-lives were, respectively, 3.5 and 6.4 h in cometabolic conditions and 9 and 19.7 h in the absence of additional carbon and energy sources. Amounts of the lipid peroxidation product malondialdehyde (MDA) and reduced glutathione (GSH) increased during NP and 4-t-OP biodegradation in cometabolic conditions by 1.7 and 2 times, respectively, as compared with a control. A high GSH level in *F. solani* 8F cells potentially implicated the metabolite in both AP biodegradation and strain resistance to oxidative stress. The study is the first to report on the NP and 4-t-OP degradation by the ascomycete *F. solani* in cometabolic conditions and in the absence of additional carbon and energy sources. The high AP degradation potential of soil ascomycetes was assumed to provide a basis for new environmentally safe bioremediation technologies for purification of soils and natural and waste waters contaminated with endocrine disruptors.

## INTRODUCTION

Long-chain alkylphenols (APs), such as octylphenols (OPs) and nonylphenols (NPs), are the most widespread endocrine disruptors and possess estrogen activity [1]. APs result from incomplete biological and abiotic transformation of respective polyethoxylates, which are nonionic surfactants and are broadly used in various industries [2]. Inadequate purification of industrial and municipal wastewaters is a main source of environmental pollution with APs [3]. To aggravate the problem, secondary effluent waste irrigation and sewage sludge pellets as alternative organomineral fertilizers are used in agriculture [4, 5]. APs have a high bioaccumulation potential and accumulate in living organisms to adversely affect the endocrine system as a primary target and to cause carcinogenic, teratogenic, and mutagenic effects [6, 7]. To prevent the negative consequences of AP contamination, it is of immense importance to develop highly efficient methods for removing toxic xenobiotics from the environment. Microbial AP degradation using pure cultures of bacteria [8–11] and fungi [12–17] or microbial consortia of benthic sediments [18] and soils [19] provides the most efficient and environmentally safe approach to AP elimination as compared with known physicochemical AP utilization methods (activated carbon filters, nanofiltration, photocatalytic oxidation, ozone treatment, and reverse osmosis) [20]. Several drawbacks of the physicochemical methods are related mostly to their high costs, a low efficiency of xenobiotic elimination, and the formation of more stable and toxic degradation products. The majority of microorganisms known as AP destructors are bacteria [8–10] and basidial fungi that produce laccase and lignin peroxidase, which play a key role in biotransformation of aromatic xenobiotics [13–21].

Ascomycete fungi (phylum Ascomycota) have attracted increasing attention in the past years because they predominate in polluted environments, are highly resistant to toxicants, and have efficient toxicant utilization processes. Known AP-degrading ascomycetes of the genera *Aspergillus*, *Candida*, *Fusarium*, *Рenicilium*, *Thielavia,* and others are capable of destructing NPs and OPs when their concentrations do not exceed 100 mg/L [14–17, 22–25]. The majority of fungal strains examined biodegrade APs in cometabolic conditions, and only few strains have demonstrated the capability of utilizing NPs and OPs as the only carbon and energy source [14–16, 22]. Fragmentary data are still available as to whether ascomycetes isolated from polluted environments are capable of destructing NPs and OPs at their high concentrations in cometabolic conditions and in the absence of additional carbon and energy sources.

The objectives of this work were to isolate ascomycetes that biodegrade NP and 4-tert-octylphenol (4-t-OP) from NP-contaminated soddy-podzolic loamy soil (Leningrad Region, Russia) and to evaluate the efficiency of AP biodegradation by the *Fusarium solani* strain 8F.

## MATERIALS AND METHODS

Micromycetes were isolated from soddy-podzolic loamy soil samples [Eutric Albic Retisol (Abruptic, Loamic, Aric, Ochric)], which were collected from a test field of the St. Petersburg State Agrarian University. In the laboratory, the soil samples were contaminated with NP at 300 mg/kg absolutely dry soil and incubated at 22 ± 2°C for 3 months. Fungal cultures were isolated from soil by a conventional dilution plating method with plating onto Czapek’s agar with 2% glucose; the medium was supplemented with 100 µg/mL streptomycin to suppress the growth of bacterial cultures.

Micromycete identification by culture and morphological features was carried out using standard methods and identification guides [26, 27]. Molecular methods based on ITS sequencing were used in addition. Fungal genomic DNA was isolated, amplified, and sequenced as described previously [28]. The ITS was amplified using the primers ITS1 (5’TCCGTAGGTGAACCTGCGG) and ITS4 (5’TCCTCCGCTTATTGATATGC). Sequencing of the amplification products was carried out on an ABI 3500xl genetic analyzer (Applied Biosystems, United States) at the Russian Collection of Agricultural Microorganisms All-Russia Research Institute for Agricultural Microbiology. A search for homologous sequences and identification were carried out using GenBank and BLAST (http://www.ncbi.nlm.nih.gov).

Technical NP (CAS: 84852-15-3) and 4-t-OP (CAS: 140-66-9) were from Sigma-Aldrich (United States).

To obtain stock cultures, spore suspensions (1–2 × 10^7^ cells/mL) were inoculated in 50 mL of Czapek’s liquid medium containing 2% glucose in 250-mL flasks and the flasks were incubated on a Certomat BS-1 shaker at 230 rpm at 25 ± 1°C for 2 days. To assess the capability of AP utilization, the stock cultures were added at a 1 : 9 ratio to 50 mL of Czapek’s liquid medium with 2% glucose (cometabolic conditions) or 50 mL of Czapek’s liquid medium (without additional carbon sources) in 250-mL flasks. APs were used as ethanol solutions and added to the culture medium to 300 mg/L. The ethanol concentration in control (without APs) and test cultures was 0.02% (v/v). An abiotic control (samples without fungal cultures) was used to assess a decrease in AP in abiotic conditions. Fungal strains were cultured in the dark on a Certomat BS-1 shaker at 230 rpm at 25 ± 1°С for up to 96 h, depending on the experimental goal. Fungal biomass was measured by a weight method.

NP and 4-t-OP were extracted from the culture medium and fungal biomass and assayed quantitatively as described previously [23, 28]. APs were assayed by HPLC on a Hewlett-Packard H 1090 instrument with a diode array detector at 278 nm with 1.2-nm resolution. The AP recovery rate was 98 ± 1%; the limit of detection was 5 µg/L.

Biodegradation (BD) of APs was calculated as BD_AP_ = 100 × (*C*_init_ *– C*_*t*_)/*C*_*t*_, where *C*_init_ is the initial AP content in the culture liquid and *C*_*t*_ is the AP content in the culture liquid at a certain incubation time point.

To assess the efficiency of AP elimination, we used the first-order kinetic model [15] ln(*C*_init_/*C*_*t*_) = *kt*, where *C*_*t*_ is the AP concentration at the time point *t*, *C*_init_ is the initial AP concentration (mg/mL), *t* is the degradation duration (days), and *k* is the elimination rate constant (days^–1^). The AP half-life (*t*_1/2_) was calculated as *t*_1/2_ = 0.693/*k*.

A total superoxide dismutase (SOD) activity assay took advantage of the SOD property to inhibit photochemical reduction of nitroblue tetrazolium, using the Beyer–Fridovich method [29] with minor modification [28]. Absorbance at 560 nm was measured using a Genesys 10 UV scanning spectrophotometer (Thermo Spectronic, United States). The results were expressed in U/g absolutely dry biomass.

The reduced glutathione (GSH) content was determined according to Gao and Tam with modification [30]. Wet fungal biomass (200 mg) was ground with 2 mL of 5% sulfosalicylic acid on ice and centrifuged at 6000 rpm for 10 min. The supernatant (0.5 mL) was combined with 0.6 mL of a reaction buffer (2 mL of 0.1 M Na-phosphate, pH 7.0, 0.1 mL of 1 mM EDTA, 0.04 mL of 0.15% 5,5-dithiobis(2-nitrobenzoic acid)).  The reaction was carried out for 2 min, and optical density at 412 nm was measured using a  Genesys 10 UV scanning spectrophotometer (Thermo Spectronic, United States). The GSH concentration was determined against a calibration curve and expressed in nmol/g absolutely dry biomass.

The malondialdehide (MDA) content was measured according to Dhindsa et al. [31] with minor modification [28].

Statistical analyses of the results were performed using the software package Statistica 10.0 (Stat Soft) and Past 4.x software (http://folk.vio.no/ohammer/past). Differences between variants were tested for significance by one-way ANOVA and Tukey’s post hoc test for normally distributed data or the Mann–Whitney *U*-test otherwise. Differences were considered significant at *p* < 0.05. Normality of data distributions and the equality of variances were evaluated by the Shapiro–Wilk and Levene tests. The results were presented as means ± standard deviation (M ± SD) as calculated from three independent replicas performed in triplicate each for each variant (*n* = 3).

## RESULTS AND DISCUSSION

Contamination of soddy-podzolic loamy soil with the endocrine disruptor NP changes the population sizes of main physiological groups of soil microorganisms and reduces the species diversity of the soil microbiome [32]. Activity shifts are additionally observed for nitrogen metabolism, carbohydrate cycle, and redox enzyme pools when soils are contaminated with NP. For example, soil cellulase activity has been found to increase by more than 30% as compared with noncontaminated soil, the increase correlating with greater population sizes of cellulose-degrading bacteria and micromycetes [33]. The NP content has been observed to decrease in NP-contaminated soil samples, the decrease being determined mostly by NP biodegradation by soil microbiota [32].

To evaluate the AP-destructing potential of mycelial fungi from soddy-podzolic loamy soil samples, the samples were contaminated with NP at 300 mg/kg absolutely dry soil in laboratory conditions, and 11 micromycete isolates were obtained. Preliminary identification of the cultures by morphological features showed that the isolates include fungi of the genera *Fusarium* (strains 1F, 3F, 7F, 8F, 9F, and 10F), *Penicillium* (strains 2F, 4-2F, and 5-2F), and *Trichoderma* (strains 4-1F and 5-1F). Ascomycetes of the genera are typical members of soil communities and are ubiquitous. The isolated strains were screened for AP degradation, and all of them were found to be capable of degrading both NP and 4-t-OP in cometabolic conditions. The degree of biodegradation within 72 h of culture was estimated at 69–95.1% for NP and 52.3–88.1% for 4-t-OP, varying among different fungal strains (Table 1).

**Table 1.** Biodegradation of NP and 4-t-OP (300 mg/L) by fungal isolates

Fungal test strain	BD_NP_ (after 72 h of culture), %	BD_4-t-OP_ (after 72 h of culture), %
1F	76.5	62.5
2F	73.2	60.3
3F	80.3	57.4
4-1F	88.0	77.4
4-2F	89.1	68.2
5-1F	69.4	52.3
5-2F	79.9	59.8
7F	72.1	63.2
8F	95.1	88.1
9F	77.2	54.5
10F	69.0	61.2

Of the 11 AP-degrading isolates, the strain 8F showed the greatest degrees of AP degradation within 72 h of culture (95.1% for NP and 88.1% of 4-t-OP, Table 1) and was selected for further experiments. Strain identification was verified by sequencing a fragment of the 18S–5.8S–28S rDNA sequence, and the 18S–5.8S–28S rDNA cluster of the strain 8F showed a high identity with its counterparts from *Fusarium solani* (Mart.) Sacc. strain PSC(R)T (96.68%), *F. solani* strain YIMPH300045 (99.76%), *F. solani* isolate F41 (100%), and *F. solani* isolate F61 (100%). Based on the morphological features and the results of sequencing the 18S–5.8S–28S rDNA fragment, the strain 8F was identified as *F. solani*.

**Fig. 1.**
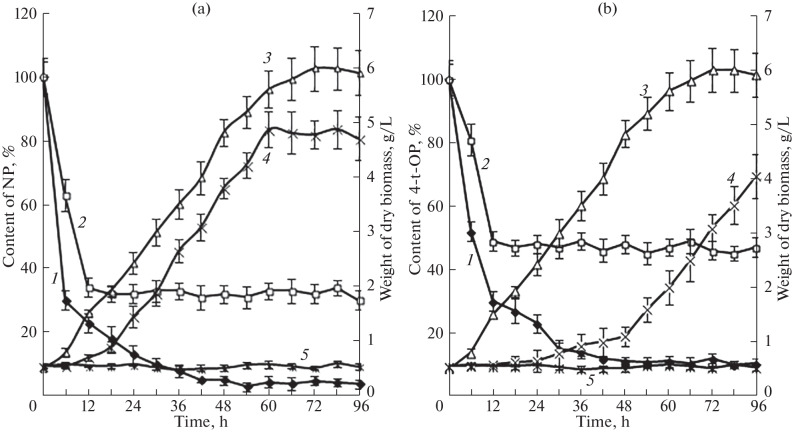
Degradation of (a) NP and (b) 4-t-OP by the *F. solani* strain 8F: *1*, elimination of AP (300 mg/L) in cometabolic conditions; *2*, elimination of AP (300 mg/L) in the absence of additional carbon and energy sources; *3*, absolutely dry biomass in Czapek’s medium containing 2% glucose (without AP, control); *4*, absolutely dry biomass in Czapek’s medium containing 2% glucose + 300 mg/L  AP; *5*, absolutely dry biomass in Czapek’s medium without glucose + 300 mg/L  AP.

The kinetic of AP degradation by the *F. solani* strain 8F was studied both in cometaboic conditions and in the absence of additional carbon and energy sources; the toxicants were used at 300 mg/L. In cometabolic conditions with glucose used as a growth substrate, APs were efficiently eliminated from the medium, although APs inhibited the growth of the test strain. In addition to the capability of degrading NP and 4-t-OP in cometabolic conditions, a metabolic property of degrading APs in the absence of additional carbon and energy sources was observed for the *F. solani* strain 8F (Figs. 1a, 1b).

An analysis of the data on AP elimination from the cultural liquid showed that biodestruction or biotransformation by the test strain and, to a minor extent, cell absorption accounted for NP and 4-t-OP elimination both in cometabolic conditions and in the absence of additional carbon and energy sources. The NP fraction bound with fungal cells ranged from 2.5 to 6.5% throughout the culture period. A somewhat higher degree of absorption, from 3.4 to 18.2%, was observed in the case of 4-t-OP.

It should be noted that a decrease in AP was not detected in the abiotic control (without a cell culture), in agreement with data from other studies [34, 35]. The NP and 4-t-OP contents in the abiotic control were 95–98% of their initial levels throughout the culture period (96 h).

A first-order kinetic model [15] was used to evaluate the efficiency of AP removal from the medium. The NP and 4-t-OP degradation kinetics of the *F. solani* strain 8F showed that degradation corresponded well to the model with the coefficients of correlation *R*^2^ ranging from 0.943 to 0.995 (Table 2).

**Table 2.** Kinetic parameters of NP and 4-t-OP elimination by the *F. solani* strain 8F

AP concentration, mg/L	Kinetic equation	Elimination rate constant *K*, h^–1^	Half-life *t*_1/2_, h	Correlation coefficient *R*^2^
^1^NP 300	ln*C* = –0.077t + 4.605	0.077	9.0 ± 0.6	0.943
^2^NP 300	ln*C* = –0.201t + 4.605	0.201	3.5 ± 0.4	0.989
^1^4-t-OP 300	ln*C* = –0.035t + 4.605	0.035	19.7 ± 1.0	0.995
^2^4-t-OP 300	ln*C* = –0.109t + 4.605	0.109	6.4 ± 0.7	0.979

^1^AP elimination in the absence of additional carbon and energy sources. ^2^AP elimination in cometabolic conditions (2% glucose).

The efficiency of AP elimination from the culture medium was compared between cometabolic conditions and conditions without additional carbon and energy sources. AP elimination was found to proceed faster in cometabolic conditions. The half-lives of NP and 4-t-OP decreased by 2.6 and 3.1 times (*p* < 0.05) to 3.5 and 6.4 h, respectively, while the AP elimination rates increased accordingly (Table 2). Both NP and 4‑t-OP were utilized almost completely (up to 95% of NP and 89% of 4-t-OP were eliminated) in cometabolic conditions, while the maximum elimination from the culture medium was 70% for NP and 52% for 4-t-OP in the absence of additional carbon sources (Fig. 1). The finding that NPs and OPs are eliminated faster in cometabolic conditions agrees with data from studies performed by other researchers [17, 22].

The NP and 4-t-OP degradation kinetics by the soil ascomycete isolates, including the most efficient destructor *F. solani* 8F, showed that the isolates are superior to all known degrading fungi in xenobiotic utilization efficiency. The fungi *Candida aquaetextoris* and *Aspergillus versicolor* have been reported to completely degrade linear 4-n-NP, which is less toxic than technical NP, within 14 and 3 days of incubation in the absence of other carbon and energy sources, respectively; the toxicant was used at 100 mg/L [22, 36]. *Candida rugopelliculosa* RRKY5 and *Galactomyces candidum* RRK17 and RRK22 isolated from sewage water have degraded 10 mg/L 4-t-OP as the only carbon and energy source within 24 h, and the 4-t-OP utilization efficiency was comparable with that observed in cometabolic conditions [14, 15]. The degradation efficiency of 30 mg/L 4-t-OP as the only carbon and energy source has been reported to range from 52 to 73% after 15 days of culture in isolates of the mycelial fungi *Fusarium falciforme*, *F. oxysporum*, *Aspergillus fumigatus*, *Trichoderma longibrachiatum*, and *T. asperellum* from sewage waters [16].

**Fig. 2.**
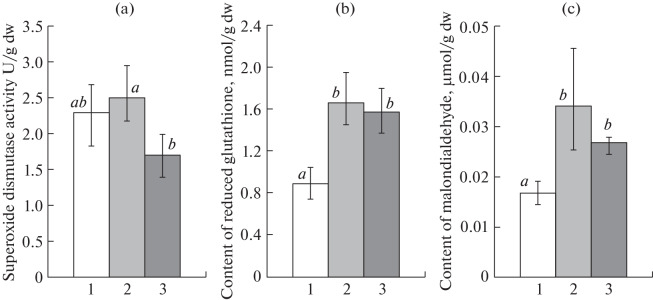
(a) SOD activity and the (b) GSH and (c) MDA contents in *F. solani* 8F cells during biodegradation of NP and 4-t-OP: 1, control (without AP); 2, 300 mg/L NP; 3, 300 mg/L 4-t-OP.

Various enzyme systems are known to play a direct role in AP degradation by mycelial fungi. The systems include copper-containing laccases, cytochrome P450 monooxygenases, superoxide dismutases, etc. [25, 37–39]. Among nonenzymatic metabolites, nonprotein peptides and, in particular, GSH attract special interest. Along with enzymatic metabolites, GSH acts as an important intracellular antioxidant and is involved in thiol–disulfide exchange, thus playing an important role in maintaining the redox status of the cell. This provides for the regulation of many cell functions, including gene expression and activities of certain enzymes and enzyme systems. Detoxification of pollutants and their metabolites is one of the main functions of intracellular glutathione, which acts both directly and as a substrate of many biotransformation enzymes [40, 41]. To study the biodegradation potential of the *F. solani* strain 8F, we analyzed the changes in protective metabolites, including SOD activity and GSH content. Compared with the control level, SOD activity did not significantly change (*p* > 0.05) during NP and 4-t-OP degradation in the *F. solani* strain 8F (Fig. 2a). In contrast, the intracellular GSH content increased significantly, by 1.7 and 2 times (*p* < 0.05), depending on the APs used (Fig. 2b).

A higher GSH level in *F. solani* 8F possibly implicates GSH in NP and 4-t-OP degradation, as well as in strain tolerance to higher AP concentrations. As is known, NP and 4-t-OP induce oxidative stress due to an accumulation of reactive oxygen species in eukaryotic cells [42, 43]. Activation of lipid peroxidation is a component of the fast stress response. MDA is a major product of lipid peroxidation and is often used as a biological indicator of oxidative stress in various organisms [44]. The MDA content in *F. solani* 8F cells increased significantly, by factors of 1.7 and 2 times (*p* < 0.05), relative to the control in the course of NP and 4-t-OP biodegradation, respectively (Fig. 2c). GSH, which possesses antioxidant properties, can act to ensure the strain resistance to AP-induced oxidative stress in this case.

## CONCLUSIONS

Fungal strains that degrade technical NP and 4-t-OP were isolated from soddy-podzolic loamy soil samples contaminated with NP. The strains were assigned to the genera *Fusarium*, *Penicillium,* and *Trichoderma*. The isolates proved capable of degrading NP and 4-t-OP at their higher concentrations (300 mg/L). The most active strain, *F. solani* 8F, degraded APs both in cometabolic conditions and in the absence of additional carbon and energy sources. APs are eliminated mostly via biodestruction or biotransformation by the strain and, to a minor extent, via absorption in fungal cells. A higher GSH content in destructor fungal cells indicates that GSH is possibly involved in xenobiotic detoxification and strain resistance to oxidative stress. A high AP degradation potential of soil ascomycetes may provide a basis for new environmentally safe bioremediation technologies for purification of soils and natural and waste waters contaminated with endocrine disruptors.
