# Erratum to: **Long-Chain Alkylphenol Biodegradation Potential of Soil Ascomycota**

**DOI:** 10.1134/S0012496623050058

**Published:** 2024-01-25

**Authors:** I. L. Kuzikova, N. G. Medvedeva

**Affiliations:** grid.513158.b St. Petersburg Federal Research Center of the Russian Academy of Sciences (SPC RAS), 199178 St. Petersburg, Russia

The article “Long-Chain Alkylphenol Biodegradation Potential of Soil Ascomycota,” written by I.L.  Kuzikova and N.G. Medvedeva, was originally published electronically in Springer-Link on October 13, 2023 without Open Access. After publication in volume 511, pages 228–234, the authors decided to make the article an Open Access publication. Therefore, the  copyright of the article has been changed to © The Author(s) 2023 and the article is forthwith distributed under the terms of a Creative Commons Attribution 4.0 International License (http://creativecommons.org/licenses/by/4.0/, CC BY), which permits use, duplication, adaptation, distribution and reproduction of a work in any medium or format, as long as you cite the original author(s) and publication source, provide a link to the Creative Commons license, and indicate if changes were made.

